# Preparing Your Manuscript: Strong and Ethical Scholarly Writing for Multidisciplinary Audiences

**DOI:** 10.1093/geroni/igab046.759

**Published:** 2021-12-17

**Authors:** Suzanne Meeks

**Affiliations:** University of Louisville, Louisville, Kentucky, United States

## Abstract

This presentation will emphasize the importance of plain, good writing. Editors read 10 or more manuscripts per week with pressure to reject 80-90% of them. If the point and contribution are not clear in a quick scan of the paper, it will not be reviewed favorably. I will provide tips for writing that are commonly violated in submissions, provide references for additional writing support, cover expectations for language consistent with GSA’s Reframing Aging initiative, and discuss some common publication ethics issues that arise during the review process, including author contributions and embedding your scholarship in the context of prior work.

